# Rapid Visual Detection of *Glaesserella parasuis* with a Real-Time Recombinase-Aided Amplification Assay

**DOI:** 10.1155/2023/9993586

**Published:** 2023-12-18

**Authors:** Haoran Kang, Dengjin Chen, Xintan Yang, Ruijiao Jiang, Cheng Song, Yongning Zhang, Lei Zhou, Xinna Ge, Jun Han, Xin Guo, Hanchun Yang

**Affiliations:** ^1^Key Laboratory of Animal Epidemiology of the Ministry of Agriculture, College of Veterinary Medicine, China Agricultural University, Beijing 100193, China; ^2^Beijing Biomedical Science and Technology Center, JOFUNHWA Biotechnology Co. Ltd., Beijing 102609, China

## Abstract

*Glaesserella parasuis* is a specific bacterial pathogen of *Glässer's* disease which causes significant economic losses to the swine industry. Dependable and rapid detection of *G. parasuis* is crucial to prevent and control *Glässer's* disease outbreaks. In this study, a recombinase-aided amplification (RAA) assay based on the infB gene was developed to rapid detect *G. parasuis*. The novel method performs isothermal detection at 42°C for real-time analysis or visualization and data analysis (RAA-VDA). The developed assay showed high specificity for *G. parasuis* detection without cross-reactions to other clinically important swine pathogens. The analytical sensitivity of real-time RAA was 67.17 copies per reaction with 95% reliability, which was comparable to the *G. parasuis* quantitative real-time PCR (qPCR). However, the detection limit of RAA-VDA was 142.43 copies per reaction with 95% reliability. The coefficient of variation analysis of the intrabatch and interbatch experimental replicate results were less than 4.30% and 6.74%, respectively, indicating the real-time RAA assay had high repeatability and reproducibility. A total of 108 clinical tissue samples were used to evaluate the clinical diagnostic performance. The diagnostic accordance rates of qPCR with real-time RAA and RAA-VDA were 100% and 98.15% (106/108), respectively. This system combined instrumental analysis and visualized analysis to accomplish a new try for rapid detection of *G. parasuis* in clinical practice.

## 1. Introduction

The Gram-negative bacterium *Glaesserella* (*Haemophilus*) *parasuis* is the pathogen of Glässer's disease in pigs and has been classified as a member of the family *Pasteurellaceae* in the genus *Haemophilus* [1]. It is an opportunistic pathogen widely colonized in the upper respiratory tract system such as the nasal cavity and trachea in healthy pigs that can cause highly fatal serositis, meningitis, bronchopneumonia, and arthritis under stress conditions [2, 3]. In recent years, under the condition of farming cluster culture, due to the expansion of breeding scale and density, *Glässer's* disease has remained a significant economic burden for the swine industry. It causes serious damage and death to pigs through coinfection or secondary infection with other pathogens, such as porcine reproductive and respiratory syndrome virus, porcine circovirus type 2, swine influenza A virus, and mycoplasma hyopneumoniae [4–7]. The high mortality and morbidity caused by the coinfection or secondary infection of *G. parasuis* pose a great challenge to the prevention and control of this disease.

According to the differences of capsular antigens, *G. parasuis* can be roughly divided into 15 serotypes, but there are still a large number of strains that cannot be typed [8, 9]. The main prevalent serotypes of *G. parasuis* in China are serotypes 4, 5, and 13 [10]. Vaccine immunization and antibiotic treatment are the main prevention and control of *Glässer's* disease. There are many serotypes of *G. parasuis*, and cross-protection between different serotypes is limited or insufficient [11]. The lack of accurate grasp of epidemic serotypes severely affects the vaccine development efforts. The current treatment of *Glässer's* disease is mainly based on the use of antibiotics but the appearance of drug resistance stains seriously reduces treatment effects.

At present, the main nucleic acid detections for the diagnosis of *G. parasuis* are PCR [12], real-time PCR [13], serotype-specific PCR [14, 15], and multiplex PCR in tissues of affected animals [16]. However, the conventional diagnostic methods mentioned above have some deficiencies, such as complicated operations, time-consuming, and requirement for complex thermal cycler instruments, which cannot meet the requirements of rapid clinical diagnosis and are not suitable for on-site real-time detection. Recombinase-aided amplification (RAA) assay is a new isothermal nucleic acid amplification technology *in vitro* with Chinese-independent intellectual property rights. This technology can achieve rapid nucleic acid amplification at a constant temperature of 37–42°C by utilizing recombinase, single-stranded binding protein, and DNA polymerase, which has the advantages of high sensitivity, strong specificity, simple operations, and no need for thermal cycler instruments [17, 18]. The amplification products can be visualized by lateral flow test strip [19, 20] and portable blue light imagers [21, 22], which is convenient for on-site real-time detection. It provides a brand-new platform for researchers to carry out in-depth research on pathogen nucleic acid detection.

**Figure 1:**
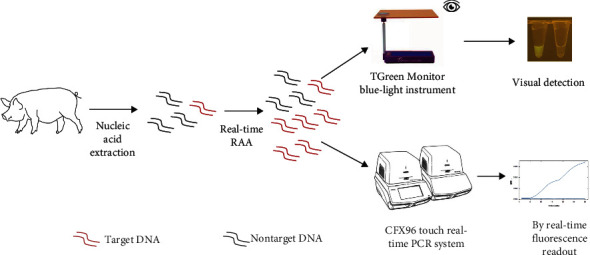
Schematic of RAA assay for detection of *G. parasuis*.

In this study, we established a RAA assay for accurate and rapid detection of *G. parasuis* in the most conserved region of infB. The diagnostic performance was compared with a TaqMan probe-based quantitative real-time PCR (qPCR) assay through clinical samples. The method can be used not only for real-time detection by probe-based fluorescence monitoring but also for visualization through portable blue-light imagers with an excitation wavelength of 480 nm (RAA-VDA) (Figure 1).

## 2. Materials and Methods

### 2.1. Bacteria Strains and Clinical Samples

The nucleic acid of *Actinobacillus Pleuropneumoniae*, *Streptococcus suis* ST171, and *Mycoplasma hyopneumoniae* was kindly provided by Dr. Bo Tang (Beijing Biomedical Science and Technology Center, JOFUNHWA Biotechnology Co. Ltd., Beijing, China); the nucleic acid of *Escherichia coli* (ATCC25922), *Klebsiella Pneumoniae* (ATCC13883), *Staphylococcus aureus* (ATCC29213), and *Pseudoonas aeruginosa* (ATCC27853) was generously provided by Researcher Yu Pang (Department of Bacteriology and Immunology, Beijing Chest Hospital, Capital Medical University, Beijing, China); the nucleic acid of *Salmonella typhimurium* (ATCC14028) was kindly provided from Prof. Jianhan Lin (China Agricultural University, Beijing, China); and the *Glaesserella parasuis* was separated and conserved in our laboratory.

A total of 108 clinical tissue samples were obtained from diseased pigs in Chinese pig farms between 2020 and 2022, including lung, lymph node, and so on. Total genomic DNA of tissue samples and bacteria was extracted using the TIANamp Genomic DNA Kit (Tiangen Biotech Co., Ltd., Beijing, China) according to the manufacturer's instructions. All extracted DNA samples were kept in collection tubes with 50 *µ*L nuclease-free water and stored at −20°C until usage.

### 2.2. Artificial Positive Control (APC)

We designed two APCs, one based on the conserved region of *G. parasuis*-infB sequences for primer screening sequence (APC1), the other based on the full length of *G. parasuis*-infB sequences for sensitivity analysis sequence (APC2). The target fragment was amplified, purified, and then inserted into pEASY-Blunt vector.

### 2.3. RAA Primers and Probe Design

Three hundred nine partial infB sequences of *G. parasuis* available in the GenBank nucleotide database were performed for multiple alignments by MAFFT software. The primers and probes were designed within the most conservative regions, following the criteria suggested in the TwistAmp™ amplification guidelines (TwistDx Ltd., Cambridge, UK).

The probe consists of an oligonucleotide backbone that contains a tetrahydrofuran (THF) residue, a flanking dT-fluorophore, a corresponding dT-quencher group, and a suitable 3′-modification group. As the internal labels used in the probe are currently only readily available on thymines, the ideal probe locations to sequences in which two thymines can be found with fewer than about five intervening nucleotides. Although several positions within the infB of *G. parasuis* meet the above criteria, an optimal one was finally selected for the design of the exo probe RAA-infB-P. Subsequently, a series of forward and reverse candidate primers were designed around RAA-infB-P using SnapGene software. The primers and probes were purchased from Tiangen Biotech (Beijing, China) and Sangon Biotech (Shanghai, China), and the detailed sequences are listed in Table 1 and Table S1.

### 2.4. Establishment and Optimization of RAA Assay

The RAA assay was performed using the kit #WLRE8208KIT of Weifang Amp-Future Biotech Co., Ltd. (Shandong, China) following the manufacturer's instructions. Briefly, each reaction contained 14.7 *μ*L of A buffer, 1 *μ*L of each forward and reverse primer (10 *µ*M), 0.3 *μ*L of probe (10 *μ*M), 2 *μ*L of nucleic acid template, 4.75 *μ*L of nuclease-free water, and 1.25 *μ*L of B buffer for the initiation of the reaction. The RAA reactions were performed in the CFX96 Real-Time Thermal Cycler (Bio-Rad, Hercules, CA, USA). Incubation was at a constant temperature of 42°C for 30 min. Furthermore, the readout results of the visual detection were based on the TGreen Monitor blue-light instrument (Tiangen Biotech Co., Ltd).

### 2.5. Real-Time PCR Assay

The qPCR assay for *G. parasuis* was performed as previously described with slight modifications [13] and was carried out using the TaqMan Fast Advanced Mix (Invitrogen, Carlsbad, CA, USA). The amplification was prepared in a final volume of 25 *μ*L containing 12.5 *µ*L of 2 × Taq Man™ Fast Advanced Master Mix (Invitrogen), 0.75 *µ*L of 10 *µ*M forward and reverse primer, 0.5 *µ*L of 10 *µ*M probe, 2.0 *µ*L of nucleic acid template, and 8.5 *µ*L of nuclease-free water. The qPCR cycling parameters initially start at 50°C for 2 min, predenaturation at 95°C for 5 min, 40 cycles at 95°C for 20 s, 55°C for 30 s, and 72°C for 30 s.

### 2.6. Analytical Specificity and Sensitivity of RAA Assay

Tenfold serial dilutions of APC2, ranging from 1.0 × 10^6^ to 1.0 × 10^0^ copies/reaction, were used to evaluate the sensitivity of the *G. parasuis*-RAA assay. For comparison, the qPCR assay for *G. parasuis* was performed in parallel with the same templates. To determine the LOD more accurately, each dilution performed eight independent reactions in both RAA and qPCR assay. Statistical analyses and data plotting were used for probit regression analysis by IBM's Statistical Product and Service Solutions (SPSS) software.

### 2.7. Repeatability and Reproducibility Analysis of RAA Assay

Three different concentrations of APC2 including high concentration (10^6^ copies/reaction), medium concentration (10^4^copies/reaction), and low concentration (10^2^ copies/reaction) were texted for the intrabatch and interbatch assays. For repeatability analysis, the APC2 was serially diluted by the real-time RAA assay and replicated three times at a time. For the reproducibility analysis, the serial dilutions were tested by the real-time RAA assay in three independent runs by different people in different times. The coefficient of variation (CV) was obtained by calculating the threshold time (TT).

### 2.8. Validation of RAA Assay by Clinical Samples

To determine the accuracy of the RAA assay, 108 clinical tissue samples were detected by the RAA assay and the qPCR assay in parallel, and the coincidence rates of the two methods were compared. Kappa statistics were used by SPSS software to determine their level of agreement. The linear regression analysis between the real-time RAA assay and the qPCR assay was performed using the GraphPad Prism software (Version 5.0; La Jolla, CA, USA).

## 3. Results

### 3.1. Screening of the Optimal Primer and Probe Combination for the RAA Assay

**Figure 2:**
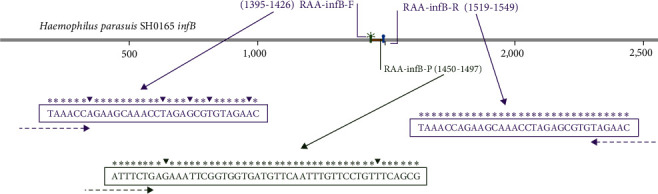
The position of the real-time RAA probe and primers within the aligned infB sequences from *G. parasuis*. The information of the sequences is listed on the left. The primer pair and probe were shaded with purple and green, respectively. Nucleotide residues that match the majority are indicated by “ ^*∗*^”. Nucleotide deletions are indicated by triangle. The dT-fluorophore residue (FAM-dT) and dT-quencher residue (BHQ1-dT) were marked by the green and blue circles, respectively.

**Figure 3:**
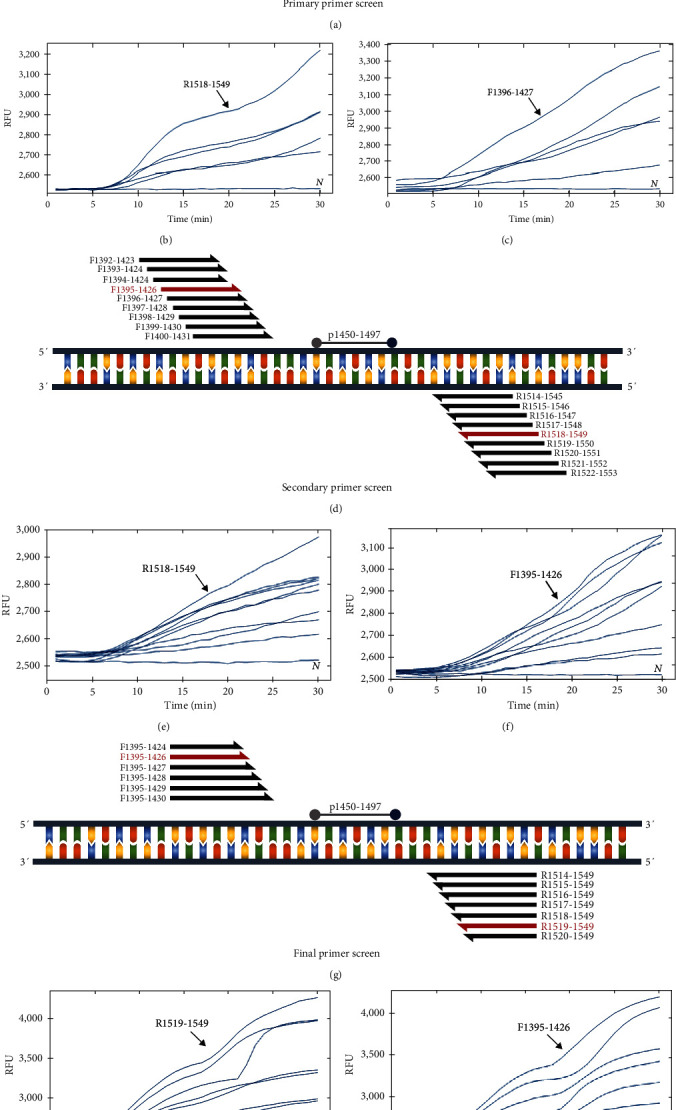
Strategies of screening primers for *G. parasuis* RAA assay. (a) Schematic diagram of the primary primer screening. The positions of the five forward and five reverse candidate primers flanking the p1450-1497 probe are denoted by arrows. The name of the primer represents the oligonucleotide positions in the infB gene of *Haemophilus parasuis* SH0165 strain. (b) The application result of the real-time RAA in the primary screening for the best reverse primer. All the candidate reverse primers were screened with the randomly selected forward primer F1372-1403. (c) The application result of the real-time RAA in the primary screening for the best forward primer. All the forward candidate primers were screened with the reverse primer R1518-1549. (d) Schematic diagram of the secondary primer screening. (e) The application result of the real-time RAA in the secondary screening for the best reverse primer. The forward primer F1396-1427 was picked to screen all reverse candidate primers. (f) The application result of the real-time RAA in the secondary screening for the best forward primer. The reverse primer R1518-1549 was picked to screen all forward candidate primers. (g) Schematic diagram of the final primer screening. (h) The application result of the real-time RAA in the final screening for the best reverse primer. All seven candidate reverse primers were screened with the best selected forward primer F1395-1426. (i) The application result of the real-time RAA in the final screening for the best forward primer. All seven candidate forward primers were screened with the best selected reverse primer R1519-1549.

In total, 309 partial sequences of the *G. parasuis*-infB gene available in the GenBank database were aligned using MAFFT software. After selecting a suitable target region, we designed an ideal exo probe that is in compliance with the criteria of TwistAmp™ amplification guidelines (Figure 2, Figure S1). First, five upstream and five downstream primers were designed surrounding the probe (Figure 3(a)). According to the screening strategy, we used a random forward primer to screen all reverse primers, picking the best reverse primer and then using it to screen all the forward primers. The key performance parameters are the time of amplification onset and the total fluorescence signal strength. After preliminary screening, the combination of F1396-1427/R1518-1549 was considered to have the best performance (Figures 3(b) and 3(c)). Subsequently, primers differing by 1 base increment around the best primary screen primers were tested in different combinations. We designed eight new forward primers around the primary screening primer F1396-1427, eight new reverse primers around the screening primer R1518-1549 (Figure 3(d)), and the combination of F1395-1426/R1518-1549 was the best primer pair through the above principle (Figures 3(e), and (f)). After having defined the best secondary screen combination, primers differing in length by 1 base increment at the 3′ end of the best secondary screen primers were tested in different combinations. We designed five new forward primers and six reverse primers around the best secondary screen combination (Figure 3(g)). Finally, the RAA detection of *G. parasuis* was performed using the optimal primer pair RAA-infB-F/R (F1395-1426/R1519-1549) with the RAA-infB-P (probe 1450-1497) (Figures 3(h) and 3(i), Table 1).

### 3.2. Analytical Specificity of the RAA Assay

**Figure 4:**
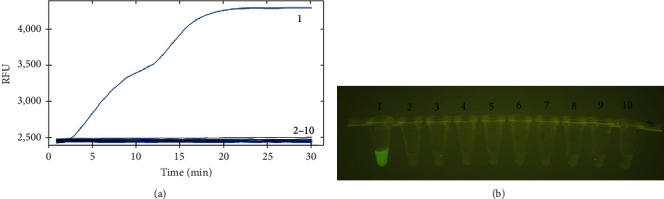
The specificity analysis of the *G. parasuis* RAA assay. Curves 1−10: nucleic acids of *G. parasuis*, *S. suis*, *K. Pneumoniae*, *A. pleuropneumoniae*, *M. hyopneumoniae*, *S. aureus*, *P. multocida*, *S. typhimurium*, *E. coli*, and negative control, respectively. (a) The specificity results of real-time RAA by real-time fluorescence readout. (b) The specificity results of RAA-VDA detection by a portable blue light imager with an excitation wavelength of 480 nm.

To determine the specificity of the RAA assay, the nucleic acids of *G. parasuis*, *S. suis*, *K. Pneumoniae*, *A. pleuropneumoniae*, *M. hyopneumoniae*, *S. aureus*, *S. typhimurium*, *E. coli*, and *P. aeruginosa* were detected by the developed RAA method (Figure 4(a)). Additionally, the amplification products can be visualized at an excitation wavelength of 480 nm by the TGreen Monitor blue-light instrument (Figure 4(b)). The assay tested positive for *G. parasuis*. There was no cross-amplification detected in the other reaction, and no signals were observed in the negative control. The results showed that the primer–probe combinations designed for the RAA assay were specific for detecting *G. parasuis*.

### 3.3. Analytical Sensitivity of the RAA Assay

**Figure 5:**
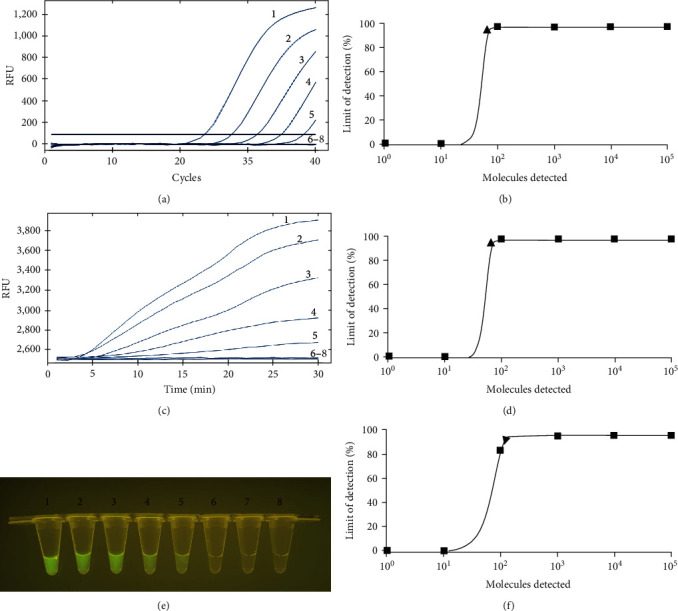
The comparison of the sensitivity with *G. parasuis* RAA and qPCR assays. Curves 1−8 : 10^6^–10^0^ copies of APC2 and negative control, respectively. (a) The sensitivity results of qPCR assay. (b) Probit regression analysis of qPCR assay with the data of eight repeats. The detection limit at 95% reliability (67.17 copies/reaction) is marked by a triangle. (c) The sensitivity results of real-time RAA assay by real-time fluorescence readout. (d) Probit regression analysis of real-time RAA assay by real-time fluorescence readout with the data of eight repeats. The detection limit at 95% reliability (67.17 copies/reaction) is marked by a triangle. (e) The sensitivity results of RAA-VDA by a portable blue light imager with an excitation wavelength of 480 nm. (f) Probit regression analysis of RAA-VDA detection with the data of eight repeats. The detection limit at 95% reliability (143.43 copies/reaction) is marked by a triangle.

The limit of detection (LOD) of the RAA method was measured using the 10-fold serial dilutions of the APC2. To generate a more accurate LOD, eight independent runs were performed for each dilution. The results of both qPCR, real-time RAA, and RAA-VDA showed that the LOD of each reaction was 100 copies (Figures 5(a), 5(c), and 5(e)). The further probit regression analysis showed that the LOD of real-time RAA and qPCR were 67.17 copies per reaction with 95% reliability (Figures 5(b), and 5(d)). Whereas the RAA-VDA demonstrated a relatively lower sensitivity, which was 142.43 copies per reaction with a probability of 95% (Figure 5(f)).

### 3.4. Analytical Repeatability and Reproducibility of the RAA Assay

To estimate the repeatability and reproducibility of the developed assay, intrabatch assay and interbatch assay CVs were determined for three concentrations of the APC. The assays were repeated three times with 10^6^ copies, 10^4^ copies, and 10^2^ copies. Based on the threshold time of the amplification, the results demonstrate that the intra-assay CV ranged from 1.64% to 4.30%, while the interassay CV ranged from 3.81% to 6.74% (Table 2). The analysis results indicate that the real-time RAA of *G. parasuis* has reliable reproducibility and repeatability.

### 3.5. Evaluation of the RAA Method by Clinical Samples

**Figure 6:**
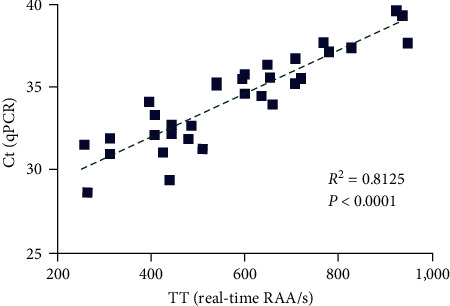
The linear regression analysis between the *G. parasuis* real-time RAA threshold time (TT) values (*x*-axis) and the *G. parasuis*-qPCR cycle threshold (Ct) values (*y*-axis) based on 33 *G. parasuis* positive samples. The analysis was performed by GraphPad Prism software and demonstrated a significant correlation between two assays (*R*^2^ = 0.8125, *P* < 0.0001).

To evaluate the diagnostic performance, 108 clinical tissue samples were simultaneously detected by RAA and qPCR. As shown in Table 3, both real-time RAA and qPCR tested 33 of the 108 samples positive for *G. parasuis*. Further linear regression analysis demonstrated a significant correlation between the two assays with an *R*^2^ value of 0.8125 (Figure 6 and Table S2). However, of the 33 positive samples tested by the real-time RAA, 31 were determined to be positive by visual observation. Therefore, the coincidence rates of qPCR with real-time RAA and RAA-VDA were 100% and 98.15%, respectively, and the kappa value between qPCR and RAA-VDA was 0.956 (*p* < 0.001) (Table 3). In summary, these results demonstrate that the developed assay can be performed on-site and has comparable clinical detection performance to the qPCR assay.

## 4. Discussion

With the expansion of large-scale agriculture in recent years, the incidence of *G. parasuis* has been steadily increasing. Currently, it has emerged as a major bacterial disease affecting the global swine industry [23]. Due to the secondary infections and coinfections of *G. parasuis* with immunosuppressive diseases, *G. parasuis* infection seriously threatens the swine industry. Although vaccines, especially autologous vaccines have been successful in reducing the mortality, vaccine failures due to poor cross-protection between different serotypes are common [24, 25]. To date, the laboratories around the world have mainly used conventional PCR and qPCR to screen and diagnose suspected cases. However, these methods have the deficiencies mentioned in Section 1, it is necessary to develop a rapid and more convenient nucleic acid detection method that can easily be used in the pig farms to detect *G. parasuis*.

In this study, we established and evaluated a conserved infB gene-based RAA method for rapid detection of *G. parasuis* in clinical swine samples. A comparison of the 309 existing *G. parasuis*-infB gene sequences in the NCBI database identified the most conserved region. The basic principle proposed by TwistDx was used to design the exo probe and screen the primers in the selected target sequence. Firstly, we found an ideal RAA-infB-P that was fully consistent with the judgment criteria and was used to screen the best primer pairs. After three rounds of primer selection, we successfully screened the most suitable primer pair RAA-infB-F/R. Then, we proceeded to evaluate its sensitivity, specificity, and repeatability. The developed RAA assay showed high specificity and had no cross-reactivity to other clinically common swine pathogens. The statistics of the intrabatch and interbatch experimental replicate results were less than 4.30% and 6.74%, respectively, indicating the real-time RAA assay had high repeatability and reproducibility. Our experimental data revealed that the real-time RAA and RAA-VDA assays have a detection limit of 67.17 and 142.43 copies with 95% reliability, respectively. In order to further evaluate the clinical diagnostic performance of this assay, 108 clinical tissue samples were detected by qPCR, real-time RAA, and RAA-VDA, respectively. The diagnostic accordance rates of qPCR with real-time RAA and RAA-VDA were 100% and 98.15%, respectively. Only two weakly positive samples were detected positive by the qPCR and the real-time RAA but negative by the RAA-VDA. It is noteworthy that the RAA products can be visualized in real time under a portable blue light imager, making it feasible to use in the field. More importantly, based on this feature, the RAA method has the potential to be exploited in a microfluidic chip technology for multiplex detection. Our results show that the established *G. parasuis* RAA is a rapid detection method with broad application prospects in the grass-roots clinical field.

As a new emerging technology with good application prospects, RAA has recently undergone steady development in the field of clinical diagnostics for animals and humans and has a broad application spectrum [26–28]. Compared with the qPCR, the real-time RAA for *G. parasuis* showed several advantages. First, the amplification process of real-time RAA can be completed within 30 min, which is much faster than qPCR. Second, RAA detection can be performed with a simple water bath and heating block, free from the requirement of expensive temperature control instruments [29]. It is noteworthy that another isothermal nucleic acid amplification technique LAMP which has a good prospect of clinical application has also been developed for the detection of *G. parasuis* [30, 31]. LAMP usually uses 4–6 primer pairs to identify distinct regions of target genes at 60–65°C for efficient amplification. Therefore, the design of primers is highly complex and technically prohibitive, and the hybridization between primers may affect the specificity of amplification [32]. Compared with the LAMP assay, the RAA assay has the following advantages. First, RAA requires only one pair of primers and is relatively easy to design [33]. Second, the RAA reaction temperature is between 37 and 42°C, which is significantly lower than that of LAMP. The energy requirement of RAA portable temperature control device is lower than that of LAMP, which is more convenient for clinical field detection. The difference between recombinase polymerase amplification (RPA) and RAA lies in the source of their recombinase. The recombinases of RPA are derived from bacteriophage T4, whereas the recombinases of RAA are derived from bacteria and fungi. Notably, RAA is a technology with Chinese intellectual property, which provides a new platform for molecular detection in China.

In summary, a real-time fluorescent RAA method targeting the infB was developed for the detection of G. parasuis. The developed method has the advantages of high specificity, high sensitivity, good repeatability, and products visualization. It can be used as a reliable tool for early and rapid diagnosis of Glässer's disease, especially in resource-limited diagnostic laboratories and clinical fields.

## Figures and Tables

**Table 1:** The primers and probes of the RAA and qPCR assays for *G. parasuis*.

Primers/probes	Sequences (5′–3′)	Size (bp)	Sources
RAA-infB-F	TAAACCAGAAGCAAACCTAGAGCGTGTAGAAC	155	This study
RAA-infB-R	CCGATTGAAGAAGAATGGCTTCAAGTAAGTC		
RAA-infB-P	ATTTCTGAGAAATTCGGTGGTGATGTTCAA(FAM-dT) (THF) (BHQ1-dT) GTTCCTGTTTCAGCG (C3-Spacer)		
CTinfF1	CGACTTACTTGAAGCCATTCTTCTT	74	[13]
CTinfR1	CCGCTTGCCATACCCTCTT		
CTinfP	FAM-ATCGGAAGTATTAGAATTAAGTGC-TAMRA		

**Table 2:** Repeatability and reproducibility analysis of the real-time RAA assay.

Concentration (copies/reaction)	Repeatability (intra-assay)				Reproducibility (interassay)		
	Mean	SD	CV (%)		Mean	SD	CV (%)
High (10^6^)	183	3.00	1.64		182	6.93	3.81
Medium (10^4^)	268	9.17	3.42		272	18.33	6.74
Low (10^2^)	644	27.71	4.30		628	36.66	5.84

Mean, the average of threshold times (second) of three independent real-time RAA reactions; SD, standard deviation; CV, coefficient of variation.

**Table 3:** Comparison of the performance of *G. parasuis* RAA and qPCR assays on clinical samples.

Assay		qPCR			Kappa	*p*-Value
		Positive	Negative	Total		
Real-time RAA	Positive	33	0	33	1.000	<0.001
	Negative	0	75	75		
	Total	33	75	108		

RAA-VDA	Positive	31	0	31	0.956	<0.001
	Negative	2	75	77		
	Total	33	75	108		

## Data Availability

Data sets used and/or analyzed during this study can be obtained from the corresponding author upon reasonable request. All data supporting this manuscript are reported and can be found in our article. Additional supporting information can be found in the online version of the article at the publisher's website.

## Supplementary Materials

Supplementary Materials Table S1: sequences of primers and probes for RAA and PCR assays. Figure S1: the aligned sequences of the target gene in *G. parasuis* infB. Table S2: the results of real-time RAA threshold time (TT) values and the qPCR cycle threshold (Ct) values.
