# Supplementary material for: Rapid Visual Detection of Glaesserella parasuis with a Real-Time Recombinase-Aided Amplification Assay
Source: Transbound Emerg Dis. 2023 Dec 18;2023:9993586. doi: 10.1155/2023/9993586 (PMC12017077; doi:10.1155/2023/9993586)
Supplement: Supplementary Materials — Table S1: sequences of primers and probes for RAA and PCR assays. Figure S1: the aligned sequences of the target gene in G. parasuis infB. Table S2: the results of real-time RAA threshold time (TT) values and the qPCR cycle threshold (Ct) values. [file 9993586.f1.docx]

Supplemental table 1：Sequences of primers and probes for RAA and PCR assays

| Primer | Sequence (5'-3') |
| --- | --- |
| F1372-1403 | GTGGTTGCGGTAAACAAAATTGATAAACCAGA |
| F1380-1411 | GGTAAACAAAATTGATAAACCAGAAGCAAACC |
| F1388-1418 | AAATTGATAAACCAGAAGCAAACCTAGAGCG |
| F1396-1427 | AAACCAGAAGCAAACCTAGAGCGTGTAGAACA |
| F1404-1434 | AGCAAACCTAGAGCGTGTAGAACAAGAGTTA |
| F1392-1423 | TGATAAACCAGAAGCAAACCTAGAGCGTGTAG |
| F1393-1424 | GATAAACCAGAAGCAAACCTAGAGCGTGTAGA |
| F1394-1424 | ATAAACCAGAAGCAAACCTAGAGCGTGTAGA |
| F1395-1426 | TAAACCAGAAGCAAACCTAGAGCGTGTAGAAC |
| F1397-1428 | AACCAGAAGCAAACCTAGAGCGTGTAGAACAA |
| F1398-1429 | ACCAGAAGCAAACCTAGAGCGTGTAGAACAAG |
| F1399-1430 | CCAGAAGCAAACCTAGAGCGTGTAGAACAAGA |
| F1400-1431 | CAGAAGCAAACCTAGAGCGTGTAGAACAAGAG |
| F1395-1424 | TAAACCAGAAGCAAACCTAGAGCGTGTAGA |
| F1395-1427 | TAAACCAGAAGCAAACCTAGAGCGTGTAGAACA |
| F1395-1428 | TAAACCAGAAGCAAACCTAGAGCGTGTAGAACAA |
| F1395-1429 | TAAACCAGAAGCAAACCTAGAGCGTGTAGAACAAG |
| F1395-1430 | TAAACCAGAAGCAAACCTAGAGCGTGTAGAACAAGA |
| R1502-1533 | GGCTTCAAGTAAGTCGTCAATCCCCATTCCTT |
| R1510-1541 | AGAAGAATGGCTTCAAGTAAGTCGTCAATCCC |
| R1518-1549 | CCGATTGAAGAAGAATGGCTTCAAGTAAGTCG |
| R1526-1557 | TAATACTTCCGATTGAAGAAGAATGGCTTCAA |
| R1534-1565 | CTTAATTCTAATACTTCCGATTGAAGAAGAAT |
| R1514-1545 | TTGAAGAAGAATGGCTTCAAGTAAGTCGTCAA |
| R1515-1546 | ATTGAAGAAGAATGGCTTCAAGTAAGTCGTCA |
| R1516-1547 | GATTGAAGAAGAATGGCTTCAAGTAAGTCGTC |
| R1517-1548 | CGATTGAAGAAGAATGGCTTCAAGTAAGTCGT |
| R1519-1550 | TCCGATTGAAGAAGAATGGCTTCAAGTAAGTC |
| R1520-1551 | TTCCGATTGAAGAAGAATGGCTTCAAGTAAGT |
| R1521-1552 | CTTCCGATTGAAGAAGAATGGCTTCAAGTAAG |
| R1522-1553 | ACTTCCGATTGAAGAAGAATGGCTTCAAGTAA |
| R1514-1549 | CCGATTGAAGAAGAATGGCTTCAAGTAAGTCGTCAA |
| R1515-1549 | CCGATTGAAGAAGAATGGCTTCAAGTAAGTCGTCA |
| R1516-1549 | CCGATTGAAGAAGAATGGCTTCAAGTAAGTCGTC |
| R1517-1549 | CCGATTGAAGAAGAATGGCTTCAAGTAAGTCGT |
| R1518-1549 | CCGATTGAAGAAGAATGGCTTCAAGTAAGTCG |
| R1519-1549 | CCGATTGAAGAAGAATGGCTTCAAGTAAGTC |
| R1520-1549 | CCGATTGAAGAAGAATGGCTTCAAGTAAGT |
| APC2-F | CAGACGCACCGAAGAAATTAAG |
| APC2-R | TCGAACGTTTAATCTCAACCAC |
| APC1-F | GGTGCATATCACGTTGAAACCGA |
| APC1-R | TACCGCACTTAATTCTAATACTT |

Nucleotide positions of primers and probes are based on the infB sequences of Haemophilus parasuis SH0165 strain.

Supplemental table 2：The results of RT-RAA threshold time (TT) values and the qPCR cycle threshold (Ct) values

| Serial number | TT（RT-RAA）/seconds | Ct（qPCR） |
| --- | --- | --- |
| 1 | 444 | 32.72 |
| 2 | 444 | 32.22 |
| 3 | 540 | 35.15 |
| 4 | 258 | 31.54 |
| 5 | 312 | 31.94 |
| 6 | 408 | 33.34 |
| 7 | 312 | 30.99 |
| 8 | 540 | 35.32 |
| 9 | 426 | 31.09 |
| 10 | 264 | 28.66 |
| 11 | 480 | 31.89 |
| 12 | 486 | 32.72 |
| 13 | 438 | 29.41 |
| 14 | 510 | 31.3 |
| 15 | 654 | 35.62 |
| 16 | 396 | 34.15 |
| 17 | 444 | 32.65 |
| 18 | 408 | 32.14 |
| 19 | 720 | 35.57 |
| 20 | 600 | 34.64 |
| 21 | 708 | 35.28 |
| 22 | 600 | 35.82 |
| 23 | 948 | 37.69 |
| 24 | 594 | 35.55 |
| 25 | 636 | 34.51 |
| 26 | 828 | 37.45 |
| 27 | 708 | 36.77 |
| 28 | 648 | 36.39 |
| 29 | 936 | 39.37 |
| 30 | 924 | 39.68 |
| 31 | 780 | 37.14 |
| 32 | 768 | 37.76 |
| 33 | 660 | 33.98 |


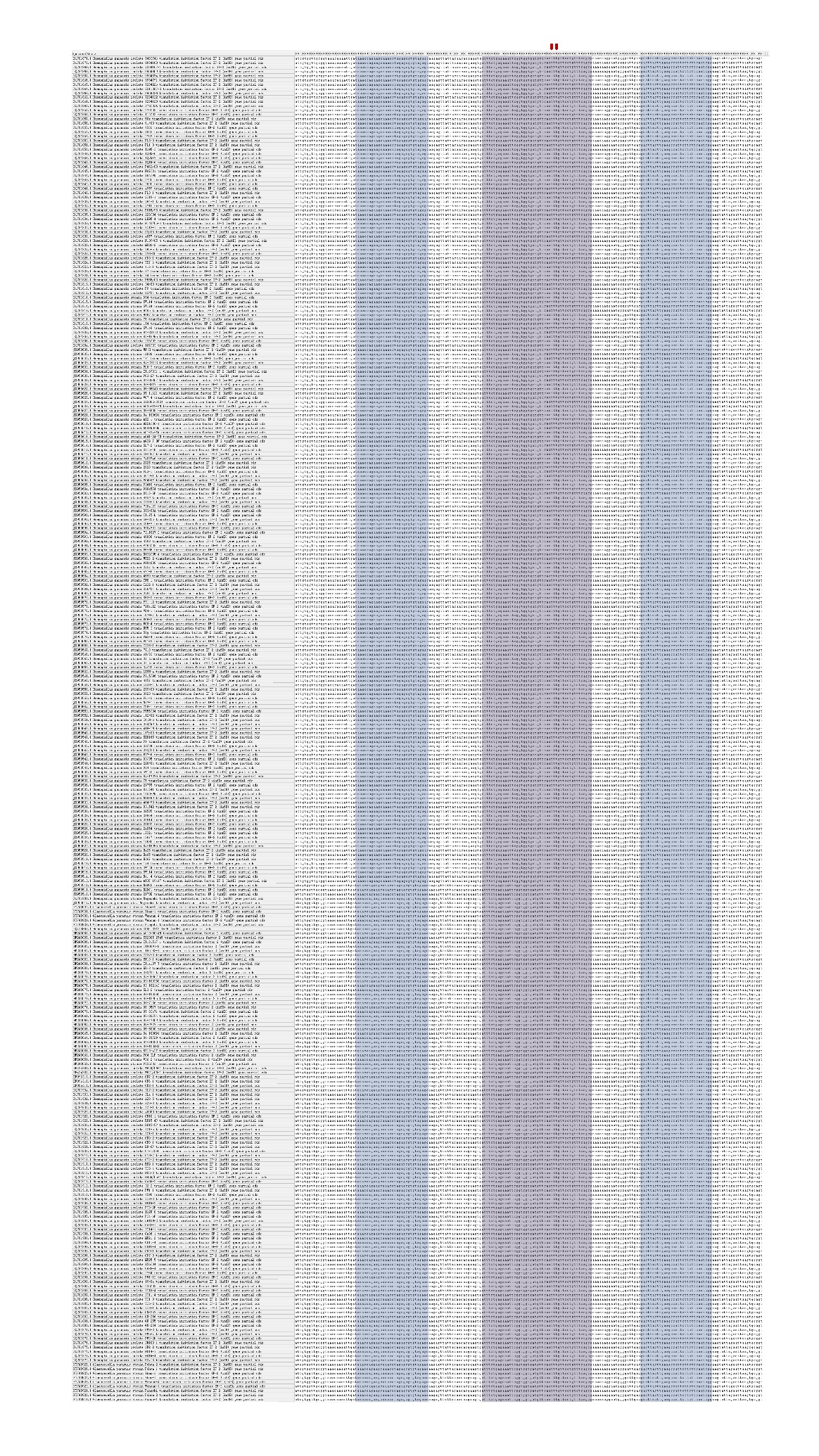


**Supplemental figure 1: The aligned sequences of the target gene in GPS-infB**

The information of the sequences is listed on the left. The primer pair and probe were shaded with blue and purple, respectively. The dT-fluorophore residue (FAM-dT) and dT-quencher residue (BHQ1-dT) were marked by the red arrows.
